# Delineating implicit and explicit processes in neurofeedback learning

**DOI:** 10.1016/j.neubiorev.2020.09.003

**Published:** 2020-11

**Authors:** Santiago Muñoz-Moldes, Axel Cleeremans

**Affiliations:** a Consciousness, Cognition and Computation group, Center for Research in Cognition & Neuroscience, Faculty of Psychology and Education, Université Libre de Bruxelles, 1050 Brussels, Belgium; b Department of Psychology, University of Cambridge, Cambridge, United Kingdom

**Keywords:** neurofeedback, biofeedback, learning, training, awareness, discrimination, consciousness, self-evaluation, performance monitoring, metacognition

## Abstract

Neurofeedback allows humans to self-regulate neural activity in specific brain regions and is considered a promising tool for psychiatric interventions. Recently, methods have been developed to use neurofeedback implicitly, prompting a theoretical debate on the role of awareness in neurofeedback learning. We offer a critical review of the role of awareness in neurofeedback learning, with a special focus on recently developed neurofeedback paradigms. We detail differences in instructions and propose a fine-grained categorization of tasks based on the degree of involvement of explicit and implicit processes. Finally, we review the methods used to measure awareness in neurofeedback and propose new candidate measures. We conclude that explicit processes cannot be eschewed in most current implicit tasks that have explicit goals, and suggest ways in which awareness could be better measured in the future. Investigating awareness during learning will help understand the learning mechanisms underlying neurofeedback learning and will help shape future tasks.

## Highlights

• This article discusses the role of awareness in neurofeedback (NF) learning.
• Most implicit NF tasks include explicit goals.
• Explicit processes are central in skill acquisition and goal-directed behavior.
• Measures of awareness are suggested to go beyond verbal exit questionnaires.
• Considering awareness is vital for understanding the learning mechanism of NF.

## 1 Introduction

Neurofeedback is a method by which a person receives information from its own brain activity, thereby potentially producing lasting neural and behavioral changes (Kamiya, 1962; Sitaram et al., 2017; Weiskopf et al., 2003). Neurofeedback can be used with a wide range of neuroimaging tools (from components of the encephalogram to the more recent development of functional magnetic resonance imaging (fMRI) and functional near-infrared spectroscopy (fNIRS)-based neurofeedback signals), different approaches to analyzing the signal (e.g., mean amplitude, multivariate patterns, etc.), diverse feedback channels (e.g., visual representations such as thermometer scales (Krause et al., 2017), but also other sensory modalities), and with different routines in the instructions given to learners. Recently, methods have been developed to use neurofeedback without the participant’s knowledge (implicitly), prompting a theoretical debate on the learning mechanisms underlying this type of learning and the necessary role of awareness.

In this article, we will seek to present a clarified understanding of the role of awareness (the state of being conscious of something) in neurofeedback learning. Awareness is notoriously difficult to define for consciousness is a mongrel (Zeman, 2005) concept that encompasses different aspects of information processing, in particular (1) phenomenal experience (i.e., “what it is like” to find oneself in a given mental state), (2) our ability to act upon information that we are conscious of, and (3) our ability to report on decisions and to intentionally monitor, control, and judge such decisions (i.e., metacognition). Because of the underlying conceptual and epistemological issues, the measurement of awareness constitutes a true challenge (see, e.g., Michel, 2017; Timmermans and Cleeremans, 2015, for reviews).

It is important to note also that people may be aware of different aspects of an experimental situation. As Nisbett & Wilson (1977) wrote, “Subjects are sometimes (a) unaware of the existence of a stimulus that importantly influenced a response, (b) unaware of the existence of the response, and (c) unaware that the stimulus has affected the response” (p. 231). Likewise, in the neurofeedback literature, *awareness* may be used to refer to different aspects of information processing: awareness of internal sensations (Brener, 1977; Frederick, 2016), awareness of being trained (Ramot et al., 2016), awareness of intended action (Ramot et al., 2016), or awareness of the relationship between a feedback signal and a specific mental content (Shibata et al., 2019).

For the purposes of this article, we will operationally define awareness as availability for report and voluntary control of action (Block, 2007). In other words, if people can talk about a particular state of affairs or intentionally act based on it (i.e., answer questions about it or make decisions about it that they can properly motivate), then we assume they are aware of it, and that they are unaware otherwise.

While cognitive processes themselves are typically not available for report (e.g., we do not consciously experience the mechanisms through which a memory is retrieved), their products and some of their characteristics may or may not be available for report. For instance, memory is deemed explicit when people report that they consciously experience the fact that they have seen the retrieved item previously, as in recall, and implicit otherwise, as when a retrieved memory can influence behavior (e.g., through priming) despite people failing to consciously recollect having seen the item previously. In such cases, however, people may experience a feeling of *familiarity* that is not linked to a specific episode. It is often the case that particular tasks, such as recognition, for instance, may involve both explicit and implicit processes: I may recognize an item because I know that I have seen it before, or merely because it feels familiar and I guess it is likely that I have seen it previously.

Finally, during learning, people may be consciously attempting to control their behavior so as to improve their performance, or they may not. Compare for instance the activity of reading a novel with the activity of attempting to memorize it when studying for an exam. The latter is characterized by intentional attempts to memorize the text and is a type of explicit, intentional learning that involves cognitive control and the self-monitoring of learning success. The former, in contrast, lacks intention (i.e., intention to commit the material to memory), cognitive control and self-monitoring. While such implicit learning might well result in declarative memory of certain elements of the material, such learning is best described as incidental, that is, as a mere side-effect of processing rather than as the core intentional goal of the activity.

With these considerations in mind, different questions arise with respect to neurofeedback paradigms. For instance, is learned control dependent on participants being consciously and intentionally engaged in self-regulation? To what extent are distinct learning mechanisms targeted with different neurofeedback paradigms? We surmise that answering these questions is important for several reasons: first, to elucidate which mechanisms are at play in neurofeedback learning, thereby advancing our basic understanding of the underlying mechanism; second, to refine the paradigms that we use and potentially adapt them to target or incentivize a type of learning, and lastly, to be able to better segue the results of animal and human studies (Lovibond & Shanks, 2002).

Several different theories from different backgrounds have been proposed to explain the mechanisms underlying neurofeedback learning, such as neural conditioning (Shibata et al., 2019), system control theory (Ros et al., 2014), two-process theory (Gaume et al., 2016) or motor skill learning (Birbaumer et al., 2013) (for more comprehensive reviews, see Gaume et al., 2016; Sitaram et al., 2017; Strehl, 2014). With regards to the involvement of conscious processes, we believe most theories can be assigned to two main approaches. The first approach consists of depicting neurofeedback learning as a form of active learning in which the organism *uses* the information given to him or her to perform voluntary mental actions towards a goal (e.g., using some cognitive task to regulate his or her brain activity). Under this view, cognitive aspects such as attention, awareness, and motivation play a central role. The second approach eschews the role of high-level cognitive processes and is instead focused on characterizing learning as an outcome of repeated stimulus-response pairings, resulting in the reinforcement-driven strengthening or weakening of associations between brain signals and feedback signals (Birbaumer et al., 2013; Shibata et al., 2019).

This second perspective thus underpins the widespread idea that learning through neurofeedback can take place independently of awareness, thus functioning as a kind of implicit learning (Amano et al., 2016; Birbaumer et al., 2013; Shibata et al., 2019). The debate surrounding the role of awareness in neurofeedback learning has gained major relevance with the recent development of so-called implicit neurofeedback paradigms (deBettencourt et al., 2015; Ramot et al., 2016; Watanabe et al., 2017), and the criticism that learning in explicit neurofeedback paradigms is contaminated by placebo, experimenter bias and demand effects (Thibault et al., 2018, 2016, 2015).

The question has been explored in the larger literatures of associative learning and motor learning. These literatures have explored the role of awareness in the production of conditioned responses (Lovibond and Shanks, 2002; Mitchell et al., 2009) or in the acquisition of motor skills (Stanley and Krakauer, 2013). We will first briefly summarize views in those literatures (section 2), before discussing awareness in biofeedback and neurofeedback more specifically (sections 3 and 4).

## 2 Awareness in human associative and motor learning

Describing learning in terms of stimulus-response associations has a long history in psychology. Traditional learning theories from the first half of the twentieth century, developed by psychologists such as Thorndike or Hull, described **instrumental learning** (the learning between actions and outcomes) in terms of stimulus-response bonds that were strengthened or weakened by reinforcement (Hull, 1943; Thorndike, 1911; Yin and Knowlton, 2006). This approach aimed at studying learning by focusing on measurable inputs and outputs, free from concepts such as goals, representations, and so on that its proponents considered to be unscientific. In animal research, in particular, associative learning is often assumed to be detached from higher-order cognitive processes (Lovibond and Shanks, 2002).

However, even if simple contiguity can result in learning and is perhaps at the source of a primitive form of associative learning already present in many vertebrates and mammals (Bekinschtein et al., 2011; Heyes, 2012; Macphail, 1982), it is often considered neither sufficient nor necessary for conditioning. Associative learning can instead be viewed as a more complex process, mainly driven by the acquisition and maintenance of internal representations of events that lead to predictions about the state of the world (Rescorla, 1988). In humans, the bulk of the evidence indeed supports the view that learning is often accompanied by contingency awareness (knowledge of the relationship between events) (Lovibond and Shanks, 2002; Mitchell et al., 2009), as evidenced by a recent meta-analysis and systematic review that found no evidence for fear conditioning without contingency awareness in humans (Mertens et al., 2020). Our aim here is to reiterate that delineating implicit from explicit components of learning is not trivial and requires careful theoretical and methodological discussion before one type of learning is dismissed. Current views of behavior have reestablished the importance of outcome expectancy, and inference-based neural learning systems that encode causal relationships between sensory events: anticipation and intentionality are now seen as dimensions that can be measured and manipulated (Yin and Knowlton, 2006).

Describing neurofeedback learning as *implicit learning* perhaps arises from espousing two assumptions: that neurofeedback learning is a form of **motor skill learning** (Birbaumer et al., 2013), and that motor learning is an implicit process. It is indeed clear that motor movements can be executed without awareness, and that we sometimes act without previous conscious intention (e.g., habits) (Dienes and Perner, 2007). But as Krakauer and colleagues point out, it is important to distinguish between motor execution and motor learning (Krakauer et al., 2019; Stanley and Krakauer, 2013). Motor learning research in humans has evidenced an involvement of both implicit and explicit processes: explicit processes in motor skill learning occur at a faster time-scale, require more time to unfold, and are sensitive to instruction and changes in reward contingencies. Implicit processes are considered to be error-driven, to act in parallel at a slower timescale, and to unfold faster (Huberdeau et al., 2015). Experimental manipulations, such as using verbal instructions or delaying feedback, allow to disentangle the contributions of explicit and implicit processes at different time points of learning (Schween et al., 2014; Taylor and Ivry, 2011), and have shown that explicit processes dominate during the initial learning phases (Taylor et al., 2014). Here also, our main argument is that considering neurofeedback as a form of motor skill learning should not lead to automatically dismiss the involvement of explicit processes.

## 3 Awareness in biofeedback learning

Neurofeedback can be considered in the context of the biofeedback literature, which treated the feedback-mediated acquired control of diverse physiological responses, such as galvanic skin conductance, skin temperature, breathing or cardiovascular responses. Research in this field was already interested in the link between awareness and control, in particular by identifying which physiological responses could potentially be controlled, by analyzing the relationship between discrimination ability and control performance, and by employing manipulations of knowledge of contingencies during learning. Two main theories of biofeedback learning have directly addressed the role of awareness: Awareness theory and Dual-Process Theory.

### 3.1 Awareness Theory

Awareness theory (also referred to as Discrimination theory), postulated that awareness of a physiological response was necessary for its voluntary control (Brener, 1977; Brown, 1971; Plotkin, 1981). Brener & Jones (1974) suggested that by repeated exposure to external biofeedback signals participants learned to identify and discriminate components of their experience, such as subtle physiological sensations, and to map them to changes in the external feedback signal, thus allowing them to improve their self-regulatory control. Here, by providing externalized information of internal states that do not usually surpass the threshold for awareness, biofeedback is seen as enabling the identification of those subtle sensations and as serving as a “tool for self-investigation” (Zolten, 1989). It is therefore seen as form of sensory substitution, similar to how deaf person would use tactile and visual feedback to learn to speak (Frederick et al., 2016). The theory predicts that regulation is correlated with discrimination ability, that discrimination is sufficient for control, and that regulatory actions become, with training, increasingly refined to physiological subsystems. However, in contradiction with the theory, control is not always associated with discrimination ability (Lacroix and Gowen, 1981), as further discussed in section 4.

### 3.2 Dual-Process theory

The Dual-Process theory (Dunn et al., 1986; Lacroix, 1986, 1981) posited that biofeedback learning is governed by both efferent and afferent (feedback-driven) processes. It claimed that neurofeedback learning consisted mostly of the former, occurring at the “central”, conscious, level. During learning, the learner starts actively applies potential strategies to reach a goal, and the biofeedback signal allows the learner to identify, and confirm, the appropriate cognitive strategies that regulate the feedback signal. In some cases, when candidate strategies for self-regulation appear ineffective, then the above-described efferent processes might be put aside in favor of a feedback-driven process, similar to Awareness Theory, described above (see 3.1). In this case, the learner will switch to the monitoring of internal interoceptive processes and will aim to identify a correlation with the biofeedback signal. If an association can be established, then the learner will try to guess what system it corresponds to, and will again switch back to an efferent strategy by selecting a verbal library to control said response. This theory best fits to paradigms in which the participant is engaged in active self-regulation and is given a goal. However, studies have shown that self-regulation can occur implicitly, without consciously applied self-regulatory strategies (see Section 4).

## 4 Current views on the role of awareness in neurofeedback learning

With neurofeedback, there are several reasons why one might dismiss the role of awareness during learning. First, verbal reports of cognitive strategies during neurofeedback performance show no consistency between participants (Kübler et al., 2001; Neumann et al., 2003; Shibata et al., 2019), and one study found no consistent relation between verbal reports of strategies and improvements in neurofeedback performance (Kober et al., 2013). Second, the instructions given to participants for the purpose of self-regulation are not always useful for learning, and participants sometimes show better performance when not using the instructed strategies (Lacroix and Roberts, 1978; Sepulveda et al., 2016; see Paret et al., 2019 for a recent review and discussion on use of instructions). Third, learning seems to be possible without awareness of neurofeedback, that is, in paradigms where the participant has no explicit knowledge of the relation between his or her neural activity and the feedback, while still being able produce neural changes with neural location specificity (Amano et al., 2016; Shibata et al., 2011). In addition, learning seems to be possible with passive neurofeedback setups, where there is no apparent goal (e.g., passive settings, as opposed to aiming to maximize a reward), suggesting that goal-directedness is also not necessary for learning (Ramot et al., 2016). Finally, learning is possible in other non-human animals (rodents, rabbits, cats, etc.), which for some authors is indicative that the learning process at play is implicit (Birbaumer et al., 2013).

These views are explicitly expressed in recent reviews of neurofeedback. For instance, Birbaumer and colleagues claimed that even though participants were using “imagery and other abstract cognitive activities” and were motivated by instruction, the “brain responses are learned, stored, and retained in a manner that is comparable to motor skill, following the rules of implicit learning” (Birbaumer et al., 2013, p. 298). They suggest that complex cognitive activities allow for the neural activity to reach a certain pattern, which then becomes reinforced by an implicit learning mechanism. The authors point to the involvement of cortical-basal ganglia loops (Birbaumer et al., 2013), which are also involved in implicit learning, and infer from there that learning is implicit. Other authors also argued in favor of learning processes that do not require awareness in the context of tasks that do not provide initial instructions (Shibata et al., 2019; Watanabe et al., 2017). In this type of task, which the authors call implicit neurofeedback, crucially, the signal itself is explicit, participants have explicit knowledge about the contingency relationship between feedback and their brain activity, and are instructed to self-regulate (as described by the authors: “participants are merely asked to make an effort to achieve better scores” (Shibata et al., 2019, p. 540)). However, the authors explain, the participants do not know “the purpose of the experiment, how the criterion has been determined or how to match induced fMRI signals to the criterion." (Shibata et al., 2019, p. 540). In addition, with these so-called implicit tasks, exit questionnaires seem to indicate that participants report not having used any particular strategy, leading authors to suggest the involvement of mechanisms of implicit learning. But, as we will note later, participants are still asked explicitly to “make an effort” to self-regulate a signal, so the role of explicit processes should not be simply discarded.

Here, we argue that this ongoing debate about the role of awareness in neurofeedback requires careful consideration of three dimensions: a) how awareness is measured, b) how instructions are communicated to participants and c) whether the learner is exposed to an active or to a passive learning situation. Heeding these three dimensions might help establish differences between different tasks and, at the same time, help identify which type(s) of learning are involved in each. We therefore propose a novel taxonomy of neurofeedback paradigms, as follows.

## 5 A novel taxonomy for neurofeedback paradigms

### 5.1 Explicit vs. implicit paradigms

Neurofeedback protocols are commonly divided into the explicit or implicit category (Gaume et al., 2016; Lubianiker et al., 2019). According to this taxonomy, in explicit paradigms, the participant has conscious knowledge of the origin of the feedback and is instructed to actively regulate it. In implicit paradigms, however, the participant is not aware of the contingency between his or her brain activity and the feedback, and may be asked to merely passively visualize a feedback display. It has been suggested that the latter type of paradigm is not influenced by the confounds of placebo, cognitive effort and other demands (Lubianiker et al., 2019), and might instead involve a different learning mechanism (Shibata et al., 2019).

However, we also argued that this binary categorization is often insufficient to describe common neurofeedback paradigms and the learning mechanisms they appeal to. One such example, already discussed in section 4, is the *implicit neurofeedback* commonly associated with “Decoded Neurofeedback” (Shibata et al., 2019; Watanabe et al., 2017). In this type of task, the feedback signal itself is explicit, and participants have explicit knowledge about the contingency relationship between feedback and their brain activity, and are asked to regulate it actively. We can already notice that the description of this task does not correspond to the use of “implicit” in the paragraph above (Lubianiker et al., 2019), where implicit refers to being unaware of the contingency and being in a passive learning situation.

Certainly, every task will inevitably involve conscious and unconscious processes (Jacoby et al., 1992). However, a better dissociation of the paradigms and their different aspects might allow to make better inferences about the learning mechanisms that are involved. Thus, a learning process could potentially rely on automatic and implicit mechanisms while being driven by conscious, effortful explicit processes. Given that it is not clear how attention and intention play a role in different kinds of neurofeedback paradigms, we propose a new taxonomy of neurofeedback learning tasks.

### 5.2 A four-category taxonomy

We suggest that in practice, most implicit neurofeedback tasks still involve explicit processes, and thus propose a different, more fine-grained taxonomy of paradigms, based on the following three dimensions:

1. **Active control**: being aware of the possibility to control or influence the feedback, as opposed to passive settings where there is no aim or goal: "*I know that my behavior will influence the feedback"*.
2. **Awareness of neurofeedback**: being aware of the neuro-feedback contingency, as opposed to thinking there are other reasons why the feedback is altered that are independent of the brain activity: *"I know that my brain activity influences the feedback"*.
3. **Awareness of strategy**: use of a strategy or cognitive task, obtained from verbal instruction or other contextual elements of the task, as opposed to finding one's own strategy: *"I have an idea of what I should do to influence the feedback"*.

In light of these three dimensions, we propose the following four-category taxonomy:

1 Active overt cued tasks
2 Active overt uncued tasks
3 Active covert tasks
4 Passive covert tasks

We will now overview the common neurofeedback paradigms and categorize them into the four new groups.

In the **active overt cued task**, the three dimensions (active control, awareness of neurofeedback, and awareness of strategy) are present. This task corresponds to the most extensively used neurofeedback paradigm. First, rewarding elements are present, either as primary rewards (monetary or appetitive incentives) or as secondary rewards (the feedback signal itself coupled with instructions to reach a goal, e.g., "increase the level of a thermometer-like display"). Second, the participant is informed that the feedback depends on his brain activity. Third, the participant is given, through verbal instructions or context, cues relating to a cognitive strategy to self-regulate his or her neural activity. The strategy can be more or less abstract (e.g., "think about movement" vs "think about moving your right hand wrist"), can be given through contextual cues (e.g., contextual affective induction in Zaehringer et al., 2019), can be a single strategy or a list of suggestions, and can be more or less flexible ("maintain it for the whole duration of the task" vs. "adapt it based on the feedback").

The **active overt uncued task** is similar to the one described above, except that no cues for a strategy are provided to the participant. He or she is informed that the feedback (e.g., the size of a circle) will change depending on his brain activity, and that he or she needs to find ways to change it (Cortese et al., 2017, 2016). Exit questionnaires are often used to find out what cognitive strategy (if any) the participant was using to reach the target. These questionnaires usually point to an important variability between subjects in the content of their strategies, and even in their degree of active control (trying different strategies vs. being more passive towards the feedback – examples of responses are provided in Cortese et al. (2017)), leaving the question open as to what extent active strategy use is necessary, if conscious strategies are perhaps employed but not reported, or even the possibility that strategies are used unconsciously (Shibata et al., 2019). The *active* control dimension is therefore debatable. However, one thing that is clear is that the participants explicitly know that the feedback is associated with their brain activity, as opposed to the category developed below.

**Active covert tasks** are characterized by the presence of active control, but the absence of awareness of neurofeedback and awareness of strategy. In these covert tasks, the neurofeedback is usually disguised as changes in parameters of the task, for example the contrast of an image (Gantner et al., 2010), the visibility of a composite image (deBettencourt et al., 2015), or as the degree of completion of an image puzzle (Ramot et al., 2017). The participant is not asked to remain passive: there is a goal given through instructions, for example, “attempt to reveal the puzzle” (Ramot et al., 2017) or "attend to one image category" (deBettencourt et al., 2015). Thus, participants are engaged in a goal-directed manner, but as opposed to *active overt uncued* tasks, they are not asked to actively regulate their brain activity and are not told that the feedback is guided by it. While the instructions do not inform about the presence of neurofeedback, it is possible that the association could be guessed or deduced from the context (e.g., presence of brain recording devices coupled with vague instructions). This possibility is also usually addressed by post-experiment interviews to ensure that participants have no knowledge of the neural - feedback contingency.

In **passive covert tasks**, the three dimensions are absent, as participants are not even aware that there is a presentation of feedback (Ramot et al., 2016). In this situation, there are still elements that are inherently rewarding (e.g., positive or negative sounds), but their degree of *controllability* is hidden. To our knowledge, there is only one study with neurofeedback in humans corresponding to these criteria. In that study, Ramot and colleagues (2016) used positive or negative sounds that were associated with specific patterns of activation in two different brain regions. Crucially, this information was hidden from participants, who were simply told that the goal of the study was to investigate their reactions to positive or negative sounds. Therefore, participants had no goal or aim for the task, and thus no conscious incentive to actively influence the feedback.

The new proposed taxonomy is summarized in Table 1. As laid out above, three different dimensions are proposed: active control, awareness of neurofeedback, and awareness of strategy, in order to disentangle differences in the context in which brain activity is reinforced in each respective neurofeedback paradigm.

**Table 1:** Fine-grained taxonomy of awareness in neurofeedback paradigms. The ▲ symbol indicates presence, □ indicates absence.

	Presence of a feedback signal from the brain	Active control	Awareness of neurofeedback	Awareness of strategy	Example
Active Overt Cued Neurofeedback	▲	▲	▲	▲	Most neurofeedback tasks
Active Overt Uncued Neurofeedback	▲	▲	▲	□	Cortese et al., 2017, 2016; Taschereau-Dumouchel et al., 2018
Active Covert Neurofeedback	▲	▲	□	□	deBettencourt et al., 2015; Ramot et al., 2017
Passive covert Neurofeedback	▲	□	□	□	Ramot et al., 2016

### 5.3 Intentionality in the brain-computer interface literature

In the field of brain-computer interfaces, a similar classification has been used to differentiate between *active*, *passive* and *reactive* setups (Zander and Kothe, 2011). According to this classification, in *active* BCIs, control depends upon intended actions and their direct correlates, e.g. neural activity associated with motor imagery in a motor imagery-based BCIs (Pfurtscheller and Neuper, 2001). In *reactive* BCIs, control is indirectly achieved through intended actions, but is actually driven by automatic responses to external stimulation, e.g. changes in neural activity driven by the active selection of flashing letters in a P300 speller, (Farwell and Donchin, 1988). Lastly, in *passive* BCIs, control originates from reactive responses that originate automatically from the interaction with the environment, but which do not follow intended actions (e.g. neural or other physiological responses that occur while interacting with a machine) (Zander et al., 2009; Zander and Jatzev, 2009). Our proposed taxonomy here borrows aspects of the aforementioned classification, in particular the importance of the user’s *intention* that delineates between active and passive paradigms in our taxonomy. However, we extend this classification to also take into account the possibility for awareness of the neurofeedback signal and awareness of a mental strategy.

### 5.4 Conclusion of the taxonomy proposal

This categorization we propose adds more nuance to the explicit vs. implicit distinction, in which the "explicit" or "implicit" label could be referring to different aspects of the design. While in some implicit tasks participants are not aware of the area or pattern of activation that is rewarded and what it represents, and exit questionnaires do not consistently indicate the use of a strategy, we think it is useful to point out the differences in the learning context and knowledge of contingencies with respect to covert and passive neurofeedback tasks.

## 6 Measures of awareness in neurofeedback

What options are there to measure awareness in neurofeedback tasks? What is the validity of these measures? Here, we will explore measures of participants’ discrimination of brain states. Traditionally, discrimination measures and paradigms have been used to find out whether the ability to discriminate between brain states (or other physiological states, as in the case of biofeedback) is related to the ability to regulate said states, thereby informing theories of how the two relate. Measures of discrimination can therefore be useful metrics to uncover the role of awareness of brain and body states in neurofeedback learning. But the measurement of awareness is famously difficult and can be subject to many pitfalls (for a review, see Timmermans & Cleeremans, 2015). In the following, we briefly review these subjective, objective and metacognitive measures.

Following the nomenclature in the consciousness literature, we categorize these measures as being either *subjective* (i.e., first-person data, from questionnaires or verbal reports), *objective* (i.e., third-person data obtained from forced-choices of carefully selected alternatives, such as detection or categorization tasks), or *metacognitive* (i.e., indices of the relation between objective and subjective performance).

### 6.1 Subjective measures

The most straightforward way to find out what participants are experiencing is to ask them directly. **Verbal reports** have indeed been used in neurofeedback studies to measure what participants are experiencing during self-regulation, and in particular, whether there is a relation between their experience (often resulting from the use of cognitive tasks or strategies) and their performance. For instance, Wolpaw and colleagues (1991) noted that: “Subjects reported that they adopted various strategies, such as thinking about a certain activity (e.g., lifting weights) to move the cursor down, and thinking about relaxing to move it up. As training progressed, several reported that such imagery was no longer needed.” (Kübler et al., 2001; Neumann et al., 2003). Beyond verbal reports, other subjective measures can be used to capture different aspects of subjective experience. For example, a subjective measure can consist of asking for ratings of vividness of visual imagery (ranging from trial-by-trial ratings to questionnaires at the end of the experiment, such as the VVIQ) (Cui et al., 2007; Marks, 1973). These measures resemble others that have been used for visual perception and memory, such as the Perceptual Awareness Scale (Ramsøy and Overgaard, 2004), a scale where participants rate their visual experience from “nothing” to “clear experience”, or the Feeling of warmth (Metcalfe, 1986), similarly a subjective measure where participants report a “feeling of warmth” for words in a memory task.

But verbal reports and other subjective measures don’t always tell the full story, as illustrated by studies showing the limits of human introspection (Nisbett and Wilson, 1977). One of the main limitations of verbal reports is that they are often obtained retrospectively, usually at the end of the experiment (Newell and Shanks, 2014), instead of on a trial-by-trial basis. In addition, participants are not always incentivized to give the full details of their experience, or might not think the information they have is relevant (Timmermans and Cleeremans, 2015) for the question. All these factors could lead to incomplete measures of awareness and to the conclusion that awareness is not present or unrelated to the performance. But absence of evidence is not necessarily evidence of absence. An alternative goal is to aim for measures that are exhaustive, which would capture awareness if and when it is present.

### 6.2 Objective measures

Due to the limitations of verbal reports, many researchers focused on developing more objective measures of awareness. In **third-person objective measures**, awareness is measured by making participants choose between two alternatives that have been carefully selected, in a way that performance above chance is taken as indicating that the participant possesses relevant information regarding their current mental state. For example, in the studies of Kamiya (Kamiya, 2011, 1968, 1962) participants were asked to answer ‘A’ or ‘B’ if they estimated the alpha power activity measured in their brain to be low or high, and received feedback for their discrimination accuracy. Above-chance accuracy in this case is taken as an indication that the participant is aware of his mental state (or has some relevant information) allowing him or her to indicate if alpha was low or high. Several studies have used such measures in neurofeedback, such as for slow cortical potential discrimination (Kotchoubey et al., 2002) or alpha level discrimination (Frederick et al., 2019, 2016; Frederick, 2012). Beyond dichotomous choices, Schurger and colleagues (2017) used continuous ratings for evaluating mental actions: participants rated the position of a cursor that was driven by their sensorimotor activity on a 1-10 point scale, before seeing their feedback, which allows to verify whether participant’s continuous guesses correlate with trial-to-trial performance.

But this approach can also be limited, because it assumes that awareness is related to objective behavior, which is not always the case (Timmermans and Cleeremans, 2015). The dissociation between objective performance and consciousness is famously illustrated by the “blindsight” condition, where people with V1-cortical damage show no conscious awareness of the stimuli presented to them, despite being capable of above-chance performance in detecting or discriminating the same stimuli (Ko and Lau, 2012). Another limitation is that objective measures can be influenced both by conscious and unconscious contents (“the contamination problem”). Thus, without empirical evidence of the association between performance and awareness, objective measures are also limited measures of awareness.

### 6.3 Metacognitive measures as candidate measures for awareness in neurofeedback

There are some measures that have not been used in neurofeedback tasks and would be useful for determining awareness. One such approach would be to focus on measuring the association between objective and subjective reports. A now widespread measure is to use ratings of confidence, based on the assumption that when one is conscious of something (seeing a stimulus), one has a sense of confidence about it (Rosenthal, 2019). Typically, metacognitive measures are directed to the participant’s behavior (e.g., confidence about a choice). For instance, a stimulus is shown to a participant who is asked to make a choice (e.g., indicating its presence or discriminating its category). A confidence rating is then asked to record how sure the participant is about having made the right choice.

The goal of **metacognitive measures** is primarily to dissociate between bias and sensitivity (Eriksen, 1960). Bias is the overall level of confidence (low or high), whereas sensitivity is the metacognitive accuracy, or the confidence-accuracy correlation: such as when one is more confident for correct trials, and less so for incorrect trials (Fleming and Lau, 2014). These views define awareness as the correlation between objective and subjective measures, thereby reflecting the ability to monitor one’s own performance. From a statistical point of view, the association can be computed in several ways: as an actual correlation (e.g., Pearson’s r) between performance scores and confidence scores, or with more advanced metrics (meta-d’ and Receiver Operant Characteristic (ROC) curves) (Harvey, 1997; Maniscalco and Lau, 2014), etc.

To conclude, measuring awareness can be subject to many pitfalls, and the exclusive use of retrospective verbal reports could be problematic. Using trial-by-trial simultaneous measures of confidence and simpler behavioral choices can shed light into the role of awareness in neurofeedback learning.

## 7 Conclusions

We conclude that explicit processes cannot be eschewed in most current “implicit” tasks, since participants are most often still aware of the contingency between their brain activity and the neurofeedback signal and they do receive explicit goals. We have thus proposed a novel fine-grained distinction based on knowledge of contingencies and the goal-directedness of learning. We have in addition reviewed and the methods to measure awareness in neurofeedback tasks, and have suggested new potential candidates. We suggest that researchers interested in elucidating the mechanism underlying neurofeedback learning use this taxonomy to identify the potential role of explicit and implicit processes.

## Declaration of Competing Interest

All authors declare they have no conflict of interest.
